# GRINL1A Complex Transcription Unit Containing GCOM1, MYZAP, and POLR2M Genes Associates with Fully Penetrant Recessive Dilated Cardiomyopathy

**DOI:** 10.3389/fgene.2021.786705

**Published:** 2021-11-25

**Authors:** Krista Heliö, Mikko I. Mäyränpää, Inka Saarinen, Saija Ahonen, Heidi Junnila, Johanna Tommiska, Sini Weckström, Miia Holmström, Mia Toivonen, Kjell Nikus, Julie Hathaway, Pauli Siivonen, Mikko Muona, Johanna Sistonen, Pertteli Salmenperä, Massimiliano Gentile, Jussi Paananen, Samuel Myllykangas, Tero-Pekka Alastalo, Tiina Heliö, Juha Koskenvuo

**Affiliations:** ^1^ Heart and Lung Center, Helsinki University Hospital and University of Helsinki, Helsinki, Finland; ^2^ Department of Pathology, Helsinki University Hospital and University of Helsinki, Helsinki, Finland; ^3^ Blueprint Genetics, A Quest Diagnostics Company, Espoo, Finland; ^4^ Department of Radiology, Helsinki University Hospital and University of Helsinki, Helsinki, Finland; ^5^ Faculty of Medicine and Health Technology, Tampere University, Tampere, Finland; ^6^ Heart Center, Tampere University Hospital, Tampere, Finland

**Keywords:** GCOM1, MYZAP, dilated cardiomyopathy, autosomal recessive, cardiomyopathy

## Abstract

**Background:** Familial dilated cardiomyopathy (DCM) is a monogenic disorder typically inherited in an autosomal dominant pattern. We have identified two Finnish families with familial cardiomyopathy that is not explained by a variant in any previously known cardiomyopathy gene. We describe the cardiac phenotype related to homozygous truncating *GCOM1* variants.

**Methods and Results:** This study included two probands and their relatives. All the participants are of Finnish ethnicity. Whole-exome sequencing was used to test the probands; bi-directional Sanger sequencing was used to identify the *GCOM1* variants in probands’ family members. Clinical evaluation was performed, medical records and death certificates were obtained. Immunohistochemical analysis of myocardial samples was conducted. A homozygous *GCOM1* variant was identified altogether in six individuals, all considered to be affected. None of the nine heterozygous family members fulfilled any cardiomyopathy criteria. Heart failure was the leading clinical feature, and the patients may have had a tendency for atrial arrhythmias.

**Conclusions:** This study demonstrates the significance of *GCOM1* variants as a cause of human cardiomyopathy and highlights the importance of searching for new candidate genes when targeted gene panels do not yield a positive outcome.

## Introduction

Dilated cardiomyopathy (DCM) is defined by reduced ejection fraction and dilation of the left ventricle in the absence of other causative factors such as abnormal loading conditions or severe coronary artery disease. (Elliott et al., 2008). Clinical onset is typically in adulthood but may also take place in infancy. (Hershberger et al., 2009). Estimates of DCM prevalence range between 1:250 and 1:3,000. (Codd et al., 1989; Mestroni et al., 1999; Hershberger et al., 2013). Phenotypic expression and severity vary greatly, even within the same family. (Hershberger et al., 2010). Patients typically present with advanced disease, including severe heart failure, arrhythmias, and sudden cardiac death (SCD), but the disease may also be asymptomatic while developing. (Hershberger et al., 2010). Familial DCM is a monogenic disorder primarily inherited in an autosomal dominant pattern. (Hershberger et al., 2010; Hershberger et al., 2013; McNally et al., 2013). Genetic etiology is broad with at least 50 genes associated. (McNally et al., 2013).

Arrhythmogenic right ventricular cardiomyopathy (ARVC) is a progressive myocardial disease that usually affects the right ventricle (RV), but it can also affect the left ventricle (LV), producing a DCM phenotype. (Elliott et al., 2008; Marcus et al., 2010). ARVC is characterized histologically by fibrofatty replacement of the myocardial tissue and clinically by right ventricular dysfunction, ventricular arrhythmias, and risk of SCD. (Elliott et al., 2008; Marcus et al., 2010; Basso et al., 2012). The estimated prevalence of ARVC is 1:5,000, but it is a significant cause of SCD especially in young people and athletes. (Elliott et al., 2008). The majority of ARVC pathological variants are reported with autosomal dominant inheritance patterns, and there is some overlap with DCM genetic background. (Jacoby and McKenna, 2012; Campuzano et al., 2013; Hershberger et al., 2013).

As DCM and ARVC sometimes overlap in phenotypic expression, it can be challenging to distinguish these subtypes clinically. As a term, ARVC does not fully reflect the spectrum of associated phenotypes, especially the left-dominant and biventricular disease subtypes. This has led to a change in nomenclature, and the concept of arrhythmogenic cardiomyopathy (AC) has been proposed, as it better reflects the broad spectrum of the disease. (Towbin et al., 2019; Corrado et al., 2020). Genetic testing is recommended when inherited cardiomyopathies are suspected. (Hershberger et al., 2018; Musunuru et al., 2020). In the future, genetic testing may have a bigger role in differential diagnosis and specifying the cardiomyopathy subtype. Knowing the specific subtype could help better predict the prognosis and guide clinical decision making.

**FIGURE 1:**
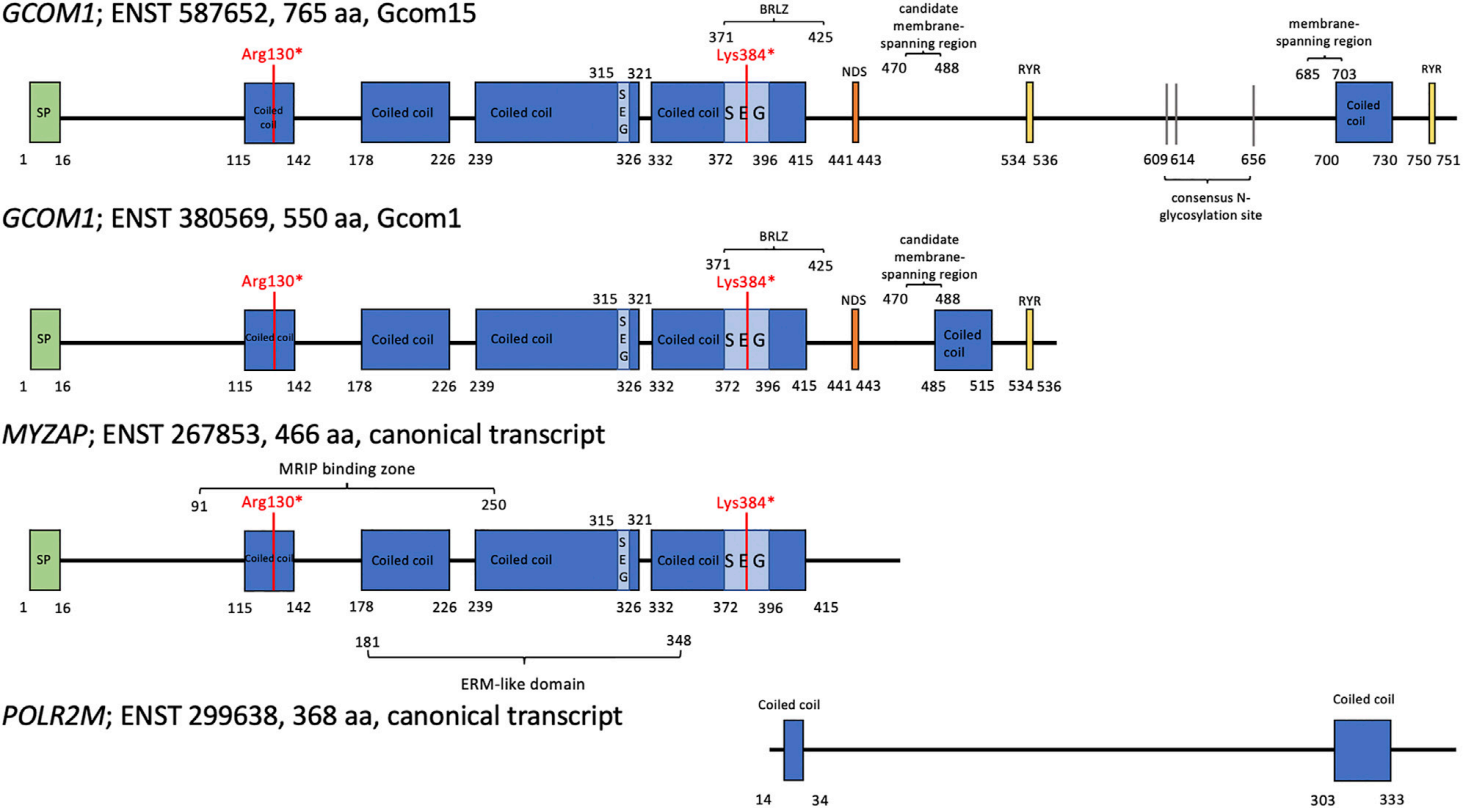
Four GRINL1A transcripts. Abbreviations: aa for amino acid, SEG for low complexity region; NDS for asparagine-linked glycosylation site; BRLZ for BRLZ leucine zipper; SP for signal peptide. The two *GCOM1* variants are marked in red; Arg130* for *GCOM1* c.388C > T, p.(Arg130*) and Lys384* for *GCOM1* c.1150 A > T, p.(Lys384*) (Roginski et al., 2004; Seeger et al., 2010; Roginski et al., 2018).

The GRINL1A complex transcription unit (CTU) in chromosome 15 comprises two neighboring genes, upstream gene *MYZAP,* and downstream gene *POLR2M*. (Roginski et al., 2001; Roginski et al., 2004; Hu et al., 2006; Seeger et al., 2010; Roginski et al., 2018). *MYZAP* encodes the protein Myozap that is mainly expressed in myocardial tissue. Seeger *et al.* noticed that a knockdown of *MYZAP* in zebrafish leads to cardiomyopathy with severe systolic dysfunction and no significant enlargement. (Seeger et al., 2010). *POLR2M* encodes the protein that is the 13^th^ subunit of RNA polymerase II. (Hu et al., 2006). The *GCOM1* combined gene utilizes exons from both *MYZAP* and *POLR2M* as well as its own exons, resulting in amino acid translations that contain residues that are not present in either of the CTU genes. (Roginski et al., 2004; Roginski et al., 2018) (Figure 1).

We have identified two Finnish families with familial cardiomyopathy not explained by variants in previously known cardiomyopathy genes. In this study, we describe the cardiac phenotype related to homozygous truncation of *GCOM1* variants in these two families.

## Subjects and Methods

### Subjects

This study included two probands who were patients at Helsinki University Hospital. All available family members were examined. All participants are of Finnish ethnicity. Clinical data were collected from all the available hospital records. Pedigrees were drawn and family history was obtained. When possible, death certificates and autopsy records were obtained. Most patients were evaluated as part of this study by 12-lead ECG, echocardiography, and physical examination. In some cases, stress-test, Holter-monitoring, cardiac magnetic resonance imaging (CMR), endomyocardial biopsy (EMB), or positron emission tomography-computed tomography (PET-CT) were performed.

DCM was diagnosed using the following criteria: systolic dysfunction (left ventricular ejection fraction [LVEF] <45%) and dilatation of the left ventricle (left ventricular end-diastolic diameter [LVEDD] >27 mm/ m^2^), in the absence of severe coronary artery disease or abnormal loading conditions such as valvular disease and hypertension. Family members were considered to be possibly affected if they had at least one of the following: atrial fibrillation (AF) at age of under years, conduction defects, left ventricular dilatation, or systolic dysfunction. In Holter-monitoring ventricular extrasystoles (VES) > 500/24 h or supraventricular extrasystoles (SVES) > 1500/24 h were considered significant. The patient with ARVC was diagnosed in the 1990s and early 2000s using the existing diagnostic criteria available at that time.

All the participants in this study gave written informed consent. This study has been approved by the Ethical Review Committee of The Department of Medicine, University of Helsinki (HYKS 26/99, HYKS 16/99, HYKS 17/99, HYKS 19/2000, HYKS 8/2000, HUS/3225/2018 Dnro 307/13/January 03, 2011, TMK11§274,December 16, 2015). Statistics Finland, Finnish Institute for Health and Welfare, and the Ministry of Social Affairs and Health have given the permission to obtain clinical data from deceased patients for research purposes (THL/1078/5.05.00/2020). This study complies with the Declaration of Helsinki.

### Molecular Genetic Studies

The genetic testing was carried out at the Blueprint Genetics laboratory at Espoo, Finland. Whole-exome sequencing (WES) was used to test both probands. Bi-directional Sanger sequencing was used to screen the familial *GCOM1* in probands’ family members. In addition, a Blueprint Genetic Comprehensive Cardiology Panel including 217 nuclear genes and mitochondrial genome was used to test I.I and I.2 of Family 1.

Mutation nomenclature is based on GenBank accession NM_001285900.3 (*GCOM1*) with nucleotide one being the first nucleotide of the translation initiation codon ATG. The pathogenicity of these *GCOM1* variants was evaluated using the American College of Medical Genetics and Genomics (ACMG) classification scheme. (Richards et al., 2015).

### Sequencing

When necessary, a bead-based method was used to extract the genomic DNA from the biological sample and electrophoretic methods were used to assess DNA quantity and quality. After quality assessment, non-contact isothermal sonochemistry processing was used to randomly fragment the qualified genomic DNA. Sequencing adapters were ligated to both ends of DNA fragment to prepare sequencing libraries. A bead-based method was used to size-select sequencing libraries to ensure the optimal template size and were amplified using polymerase chain reaction (PCR). A hybridization-based target capture method was used to target intronic targets and exons. The quality control of the completed sequencing library was made by confirming the appropriate template size and quantity and ensuring the absence of leftover primers and adapter-adapter dimers. Illumina’s sequencing-by-synthesis method using paired-end sequencing (150 by 150 bases) was used to sequence the sequencing libraries after they had passed the quality control. The sequencing instrument used Illumina’s trademarked software to carry out primary data analysis, which included converting images into base calls and associated quality scores, the final output being the generated CBCL files.

### Histology and Immunohistochemistry

Histological samples were analyzed from patients in Family 1 (II.1), and Family 2 (II.4, II.9, II.10). Other myocardial samples were used as control tissue. Samples of F1 II.4 and F2 II.4 were obtained from Helsinki Biobank.

Three μm sections were cut from formalin-fixed paraffin-embedded specimens. Antigen retrieval was done in a decloaking chamber (Biocare Medical (Decloaking Chamber, 2021)) 20 min at +95°C in EnVision FLEX Target Retrieval Solution (low pH antigen retrieval solution, Dako, product number K8005). After blocking of endogenous peroxidase with BLOXALL^®^ blocking solution (Vector laboratories, product number SP-6000–100 (Peroxidase Blocking Solution, 2021)), sections were incubated with antibody detecting MYOZAP-GCOM1 (Origene, catalogue number TA320110, final IgG concentration 0.2 μg/ ml). The antibody detects an 18 amino acid (aa) sequence near the carboxy terminus of MYOZAP (407-425aa). Dako REAL EnVision Detection System was used for visualization of the primary antibody (Dako, product number K5007). Mayer’s haematoxylin (Dako, product number S3309) was used as counter stain. Substituting primary antibody with rabbit non-immune IgG (Vector laboratories, affinity purified rabbit IgG, catalogue number I-1000 (Rabbit IgG, 2021)) at same IgG-concentration was used as a negative control for the primary antibody. Omitting primary antibody was used for ruling out unspecific staining by secondary antibody. Stained slides were scanned with PANNORAMIC 250 Flash III (3DHISTECH, Budapest, Hungary).

## Results

### Genetic Studies

We identified two homozygous truncating *GCOM1* variants, each in a different family, in patients presenting with cardiomyopathy. A homozygous *GCOM1* c.388C > T, p.(Arg130*) variant was observed in Family one in two DCM patients, and a homozygous *GCOM1* c.1150 A > T, p.(Lys384*) variant was observed in Family two in four patients with cardiomyopathy. Both variants cause a premature stop codon and are predicted to cause loss of normal protein function either through protein truncation (129/765 aa and 383/765 aa, respectively) or nonsense-mediated mRNA decay from both of the alleles.

*GCOM1* c.388C > T, p.(Arg130*) is located at exon 4/15 in the canonical transcript of *GCOM1*, but two other RefSeq transcripts of this gene (exon 4/14; NM_001018090, exon 4/13; NM_001018091) are also affected. The variant also affects exon 4/13 in *MYZAP* due to readthrough transcription. Six individuals in the Genome Aggregation Database (gnomAD, n > 120,000 exomes and >15,000 genomes) are heterozygous for this *GCOM1* c.388C > T, p.(Arg130*) variant. This variant is not reported in the Finnish reference population, and the total allele frequency is 0.00002387. *GCOM1* c.1150 A > T, p.(Lys384*) variant is located at exon 11/15 in the canonical transcript of *GCOM1* and also affects two other RefSeq transcripts of this gene (exon 11/14; NM_001018090, exon 11/13; NM_001018091). Due to readthrough transcription, this variant also affects exon 11/13 in *MYZAP*. In gnomAD, this variant is reported only in the European Finnish cohort in one heterozygous individual. Allele frequency in the Finnish population is 0.00004656, and the total allele frequency is 0.000004073. No homozygous truncating *GCOM1* variant carriers are reported in the gnomAD reference population. Individuals with severe pediatric diseases have been excluded from these cohorts by database curators.

**FIGURE 2:**
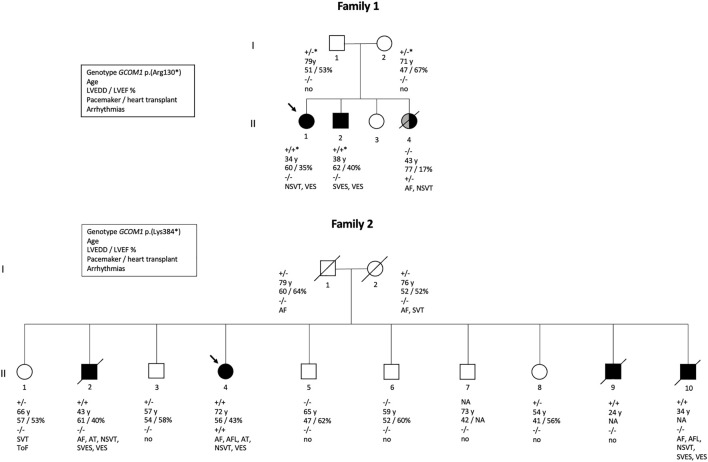
Pedigrees of two families. Family one is affected with GCOM1 c.388C > T, p.(Arg130*) variant, Family 2 with *GCOM1* c.1150 A > T, p.(Lys384). Circles represent women, squares men. Black symbols represent individuals who fulfil cardiomyopathy criteria. Grey symbol represents cardiomyopathy of other etiology. Arrow indicates proband. Genotypes: + / + homozygous and + / - heterozygous for the *GCOM1* c.1150 A > T, p.(Lys384*); + / +* homozygous and +/ −* heterozygous the for *GCOM1* c.388C > T, p.(Arg130*), − / − wild type allele. Clinical features are listed below the symbols; Age–age at the time of clinical examination or at the time of death, LVEDD left ventricular end-diastolic diameter (mm), LVEF left ventricular ejection fraction (%), pacemaker/cardiac transplant: + indicates yes,–indicates no, ToF Tetralogy of Fallot.

Altogether, *GCOM1* c.388C > T, p.(Arg130*) was detected in Family one in four individuals: two unaffected heterozygotes and two homozygotes with DCM. One family member without the variant had DCM secondary to lymphocytic myocarditis. *GCOM1* c.1150 A > T, p.(Lys384*) was detected in 11 individuals, four of them homozygotes. Two out of the four *GCOM1* p.(Lys384*) homozygotes suffered from cardiomyopathy and died at young age, one fulfilled DCM diagnostic criteria, and one received a primary diagnosis of DCM, and later of ARVC. None of the heterozygotes in either of the two families were affected. Pedigrees of families one and two are shown in Figure 2.

Of the six affected individuals with homozygous truncation in *GCOM1*, one had pacemaker (PM) and later heart transplantation, four suffered from ventricular and atrial arrhythmias, one had only ventricular arrhythmias, and one had no recorded arrhythmias but suffered SCD (Table 1). Of the six patients, three had AF or atrial flutter (AFL), four had non-sustained ventricular tachycardia (NSVT), four had VES (>500/24 h), two had SVES (>1500/24 h), and one had multiple VES and SVES in stress-test. Six of nine heterozygotes had no recorded arrhythmias, 2/9 had AF (at age >70). Two of three patients without the variant had no arrhythmias, and 1/3 had AF and NSVT. None of the heterozygotes had either PM or implantable cardioverter-defibrillator (ICD), but one family member without the variant had myocarditis-triggered DCM and received an ICD.

**TABLE 1:** The main clinical features of the probands and their relatives. Probands are marked in bold. Symbols and abbreviations: Age (M/F) age and sex (M: male/F: female); Genotype: +/ + homozygous and +/ − heterozygous for the *GCOM1* c.1150 A > T, p.(Lys384*); +/+* homozygous and +/-* heterozygous for the *GCOM1* c.388C > T, p.(Arg130*), −/ − wild type allele; NA not available; AVB1 and AVB3 for atrioventricular block 1 and 3, LAHB for left anterior hemiblock, RBBB for right bundle branch block, SSS for sick sinus syndrome; Atrial arrhythmias–arrhythmias of atrial origin: SVES for supraventricular extrasystoles >100/24 h,^†^ when observed in stress-test, AF for atrial fibrillation, AFL for atrial flutter, AT for atrial tachycardia, SVT for supraventricular tachycardia; ventricular arrhythmias–arrhythmias of ventricular origin: VES for ventricular extrasystoles >100/24 h, ^†^ when observed in stress-test, NSVT for non-sustained ventricular tachycardia; PM pacemaker, ICD implantable cardioverter-defibrillator; LVEDD left ventricular end-diastolic diameter (millimeters); LVEF left ventricular ejection fraction (%); RVEDD right ventricular end-diastolic diameter (millimeters); Age at dg–age at the time of diagnosis of cardiomyopathy; Histology: + if endomyocardial biopsy was taken, ++ if the heart was examined post-mortem or after explantation; Phenotype–phenotype at diagnosis; DCM dilated cardiomyopathy; ARVC arrhythmogenic right ventricular cardiomyopathy; Other–other significant clinical features; SCD sudden cardiac death.

Individual	Age M/F	Genotype	Conduction defect	Atrial arrhythmias	Ventricular arrhythmias	PM, ICD	LVEDD (mm)	LVEF (%)	RVEDD (mm)	Age at dg	Age at death	Histology	Phenotype	Other
Family 1														
II.1 (proband)	34F	+/+*	No	No	NSVT, VES	No	60	35	NA	24		+	DCM	Heart transplant
I.1	79M	+/−*	No	No	No	No	51	53	30					
I.2	71F	+/−*	No	No	No	No	47	67	31–33					
II.2	38M	+/+*	No	SVES	VES	No	62	40	23	37			DCM	
II.4	F	−/−	AVB3	AF	NSVT	DDD-ICD	77	17	NA	41	43	+	DCM	
Family 2														
II.4 (proband)	72F	+/+	No	AF, AFL, AT	NSVT, VES	VVIR	56	43	57	41		++	ARVC	
I.1	79M	−	AVB1, RBBB, LAHB	AF	No	No	60	64	NA		88		
I.2	76F	+/−	SSS	AF, SVT	No	No	52	52	NA		80		
II.1	66F	+/−	RBBB	SVT	No	No	57	53	NA			
II.2	43M	+/+	LAHB	AF, AT, SVES	NSVT, VES	No	61	40	72	36	55		DCM	Tetralogy of Fallot
II.3	57M	+/−	No	No	No	No	54	58	28					
II.5	65M	−/−	No	No	No	No	47	62	NA					
II.6	59M	−/−	No	No	No	No	52	60	NA					
II.7	73M	NA	No	No	No	No	42	NA	NA					
II.8	54F	+/−	No	No	No	No	41	56	NA					
II.9	M	+/+	No	No	No	No	NA	NA	NA		24	++	Affected	SCD
II.10	M	+/+	No	AF, AFL, SVES^†^	NSVT, VES^†^	No	NA	NA	NA	34	34	++	Congestive cardiomyopathy	
III.1	19F	NA	No	No	No	No	43	61	NA					
III.2	15F	NA	No	No	No	No	48	71	NA					
III.3	50F	+/−	No	No	No	No	52	60	NA					
III.4	47F	−	No	No	No	No	43	69	NA					
III.5	15M	NA	No	No	No	No	45	65	NA					
III.6	19F	NA	No	No	No	No	48	71	NA					
III.7	9F	NA	No	No	No	No	37	63	NA					

**FIGURE 3:**
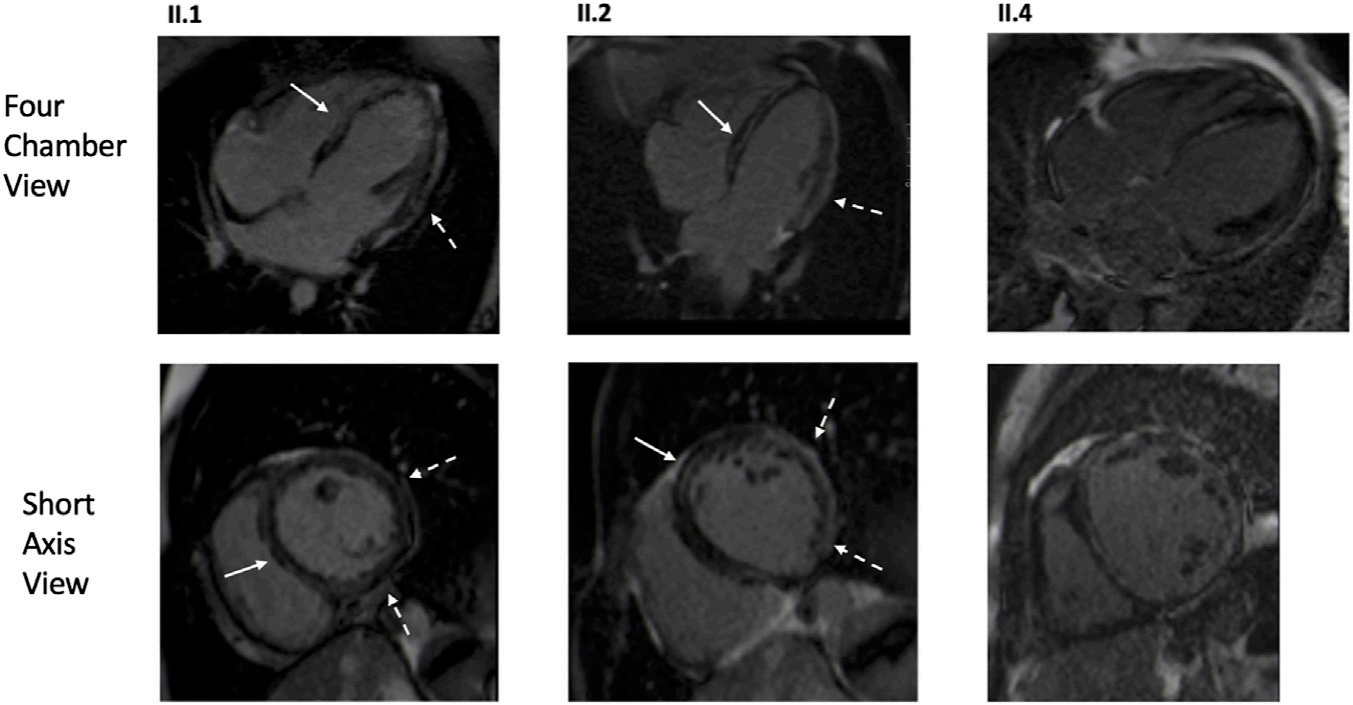
CMR images of the proband (Family 1, II.1), affected family member with homozygous *GCOM1* p.(Arg130*) (Family 1, II.2), and affected family member with no familial mutation but DCM secondary to lymphocytic myocarditis (Family 1, II.4). Both homozygotes (II.1, II.2) presented mid-wall LGE in the septum (arrows) and subepicardial enhancement in the inferolateral areas (dashed arrow). The genotype negative patient (II.4) exhibited multifocal and patchy LGE involving several layers of the myocardium.

Three patients from Family one underwent CMR: two homozygotes (II.1, II.2) and one patient without the variant (II.4) with DCM secondary to lymphocytic myocarditis (Figure 3). Both homozygotes had hypokinetic and dilated left ventricles; right ventricles were normal. Extensive LGE was observed in both; subepicardial LGE, especially in the lateral wall, and intramyocardial LGE were observed. In II.4, both ventricles were dilated and hypokinetic and extensive patchy LGE were observed. PET-CT was later performed in all three. In the homozygotes, no signs of inflammation or sarcoidosis were observed. In the patient without the variant, findings compatible with a mild inflammatory process were observed in the lateral wall and septum. On echocardiography, two patients (F2: II.2, II.4) had suboptimal left ventricular function, but also RVEDD greater than LVEDD. Other homozygotes in both families had normal right ventricular function.

**FIGURE 4:**
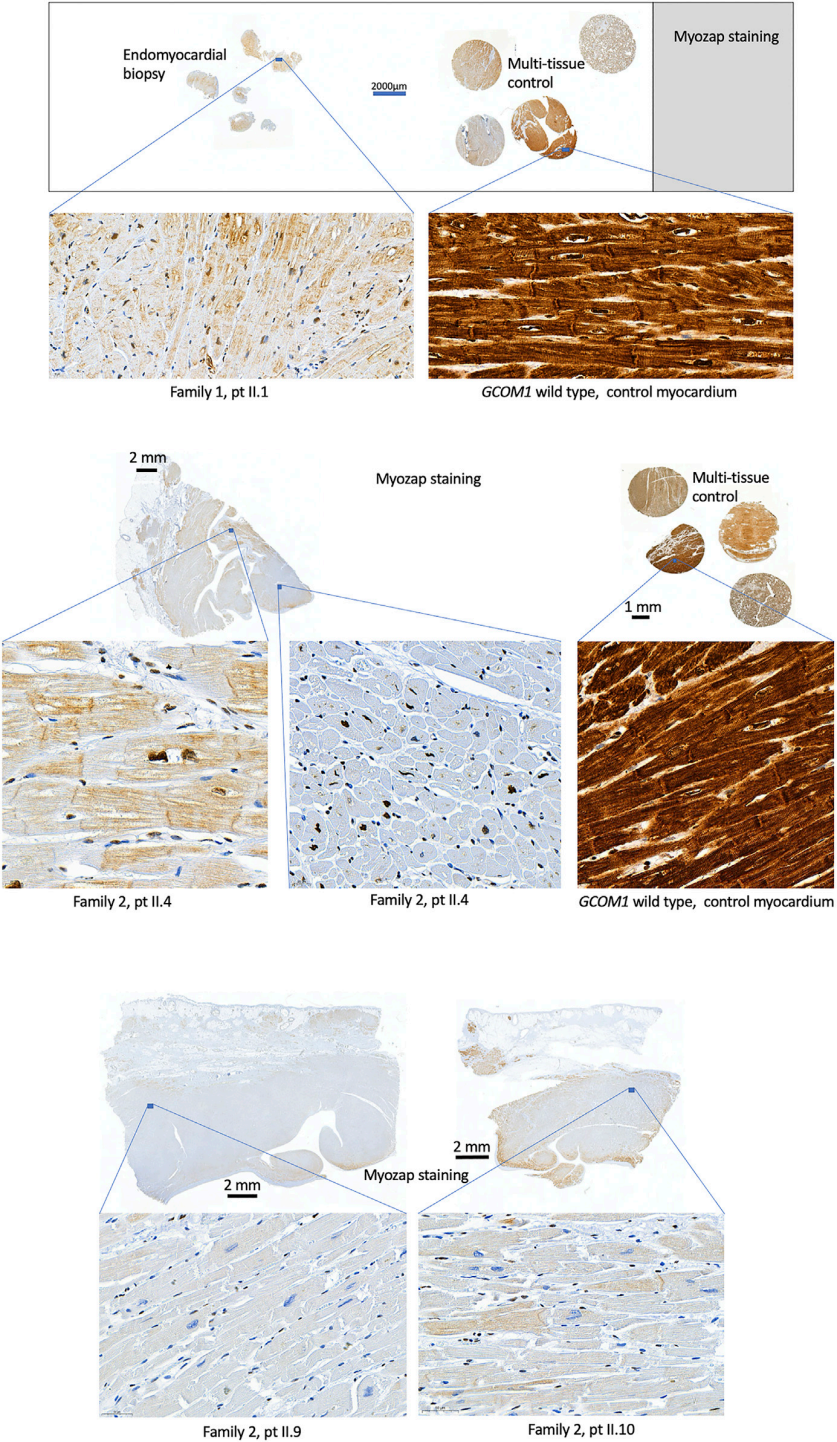
Histological samples. Myozap-staining of myocardial samples from homozygotes (Family 1: II.1 and Family 2: II.4, II.9, II.10) show clear loss of staining when compared to the wild type GCOM1 control samples. Controls with primary antibody omitted or substituted with rabbit non-immune IgG showed no staining (Data not shown).

Histological samples were available from five patients: four homozygotes (F1: II.1, F2: II.4, II.9, II.10) and one genotype negative (F1: II.4). EMB was taken from one genotype negative patient and one homozygote, the whole heart was examined post-mortem in two homozygotes, and after explantation in one homozygote. Histology indicated no signs of inflammation in any of the homozygotes, myocyte degeneration was observed in all four homozygotes and fibrosis was observed in three. The genotype negative patient had findings compatible with lymphocytic myocarditis. Fibrofatty replacement of atria, septum, and posterior wall were observed post-mortem in one homozygous patient, whereas RV was unaffected. Immunohistochemistry was conducted in the four homozygotes and wild type control samples (Figure 4). Due to poor quality and small sample size, immunohistochemistry was not conducted for the EMB of F1 II.4. Myozap-staining of the samples from *GCOM1* homozygotes showed clear loss of staining when compared to the intense brown staining in the wild-type control myocardium samples. The loss of staining demonstrates reduced expression in *GCOM1* homozygotes.

Five of the six homozygotes had a cardiomyopathy diagnosis; DCM was diagnosed at the ages of 24, 34, 36, and 37 One homozygote was diagnosed with DCM at the age of 41 but was later diagnosed with ARVC. The one homozygote who did not have a cardiomyopathy diagnosis while living experienced SCD at the age of 24 and was diagnosed with cardiomyopathy in autopsy. None of the nine heterozygous family members fulfilled any cardiomyopathy criteria. One family member (F1: II.4) without the variant had DCM secondary to lymphocytic myocarditis. Due to fragmented DNA in their sample, broad genetic testing could not be conducted. In broad panel testing, the patient’s parents (F1: I.1, I.2) were heterozygous for the *GCOM1* variant, and did not have any other potentially pathogenic variants that could explain the heart failure in the daughter (II.4), who was homozygous for wildtype alleles in *GCOM1*.

Based on the variant classification scheme that follows the ACMG guidelines, *GCOM1* c.388C > T, p.(Arg130*) and *GCOM1* c.1150 A > T, p.(Lys384*) variants can be considered likely pathogenic.

### Family 1

The proband (II.1) was a 34-year-old female who received DCM diagnosis at the age of 24. She had fluctuating Troponin I (TnI) concentrations over the years; TnI was 2,358 ng/ L at its highest (normal range <45 ng/ L). She had low voltage P-waves in an otherwise normal ECG. Holter-monitoring demonstrated NSVT and VES (1900/24 h). CMR demonstrated a dilated LV and systolic dysfunction; RV was within normal limits. In LV, EF was 38%, end-diastolic volume (EDV) was 242 ml (126 ml/ m^2^), end-systolic volume (ESV) was 149 ml (78 ml/ m^2^), and stroke volume (SV) was 93 ml. Right ventricular ejection fraction (RVEF) was 54%, EDV was 163 ml (85 ml/ m^2^), ESV was 71 ml (37 ml/ m^2^), and SV was 92 ml. Lateral wall was thin; basal thickness was around 4 mm, and apical thickness was 6 mm. Extensive circular subepicardial LGE was observed in LV, but midmyocardial involvement and patchy LGE were also detected. Segmental T1 native mapping times were slightly increased in all segments referring to diffuse myocardial fibrosis. Slight myocardial oedema was observed. In EMB, no signs of myocarditis or sarcoidosis were seen. In PET-CT, perfusion was decreased in the septum, especially in anteroseptal regions; no sign of inflammatory process or sarcoidosis were observed.

The brother (II.2) of the proband, a previously healthy 37-year-old male, was diagnosed with DCM during this study. He had been asymptomatic except for mild palpitations during stress. Holter-monitoring demonstrated VES (1028/24 h) and SVES (1545/24 h). He had low voltage P-waves in an otherwise normal ECG. TnI concentrations were elevated: 2,999 ng/ L at its highest. On CMR, LV was dilated and hypokinetic, EF was 40%, EDV was 250 ml (132 ml/ m^2^), ESV was 150 ml (79 ml/ m^2^), and SV was 100 ml. RV was normal, RVEF was 60%, EDV was 167 ml (88 ml/ m^2^), ESV was 66 ml (35 ml/ m^2^), and SV was 101 ml. Wall thickness was 7–8 mm. Segmental T1 native mapping times were increased in the inferior and lateral wall. Extensive midmyocardial LGE was observed in septum; subepicardial LGE was observed in basal and midventricular regions. Increased extracellular volume compatible with fibrosis or oedema was seen. No signs of inflammation or sarcoidosis were observed in PET-CT.

The sister (II.4) not carrying the variant had DCM secondary to lymphocytic myocarditis at the age of 41 and died at the age of 44 due to end-stage heart failure. She presented with advanced disease already at the first time she sought medical treatment. She suffered from AF and NSVT, in ECG she had prominent P-waves and a 3-degree AV-block. She later received a pacemaker. Slightly elevated and fluctuating Troponin T (TnT) levels were observed over the years (TnT 98 ng/ L, normal range <14 ng/ L). Global hypokinesia and dilated ventricles were observed in CMR. In LV, EF was 18%, EDV was 404 ml (199 ml/ m^2^), ESV was 329 ml (162 ml/ m^2^), and SV was 74 ml. In RV, EF was 28%, EDV was 266 ml (131 ml/ m^2^), ESV was 191 ml (94 ml/ m^2^), and SV 75 ml. Reduced wall-thickness was observed, 12 mm in basal septum, otherwise septal thickness was 6 mm, and inferolateral wall-thickness was only 3 mm. Left ventricular myocardium was abnormal and extensive patchy LGE was observed, transmural LGE was observed in inferolateral akinetic region and was deemed to be compatible with inflammatory process. EMBs showed severe myocyte degeneration, lymphocytes, and mast cells, compatible with lymphocytic myocarditis. PET-CT demonstrated uneven perfusion in LV walls, deficiency was also seen in apex, anterior and inferior walls, and inferior part of the septum. Findings were compatible with mild inflammatory process. She had poor compliance with the heart failure treatment. She used antipsychotic medication for over a decade for schizophrenia. Perphenazine was prescribed at the age of 33 and was later changed to risperidone, which she used for over 6 years. For 2 years she had quetiapine combined with risperidone. A year before the first cardiac symptoms, the medication was changed to paliperidone. Due to the poor sample quality, no further genetic testing could be conducted to detect possible pathogenic variants contributing to the disease.

The proband’s heterozygotic parents had normal findings in cardiac evaluations. NGS panel testing was conducted on both parents; no pathogenic/likely pathogenic or other promising variants in known cardiomyopathy genes were found. The 79-year-old father (I.1) had normal ECG. He has had some palpitations but no recorded arrhythmias. On echocardiography his LVEDD was 51 mm and LVEF was 53%. Mother (I.2) was 71 years old; her ECG was normal, and she has had no arrhythmias. On echocardiography, her LVEDD was 47 mm and LVEF was 67%. One sibling (II.3) was unavailable for the study.

### Family 2

The proband (II.4) was a 72-year-old female who received a DCM diagnosis at the age of 41 and an ARVC diagnosis at the age of 52. Echocardiography demonstrated biventricular dilatation, LVEDD was 56 mm, RVEDD was 57 mm, and LVEF was 43%. She had arrhythmias at stress and rest, Holter-monitoring demonstrated NSVT and VES (10,000/24 h). She had a tendency for atrial arrhythmias, AFL, AF, and AT. She received a pacemaker and later a heart transplant at the age of 52. Her ECG showed low-voltage P-waves and T-wave inversions in V1-V3. Her explanted heart was examined, no coronary artery disease was observed, and valves were structurally normal. RV wall-thickness was reduced to only 3–4 mm, LV wall thickness was 15 mm. On histology, thick fibrotic endocardium was observed as well as severe myocyte degeneration. There were no signs of inflammation or myocardial infarction.

One brother (II.2) received a DCM diagnosis at the age of 36 and died at the age of 55. On echocardiography wall thickness was normal, LVEDD was 61 mm, LVEF was 40%, and RVEDD was 72 mm. He suffered from presyncope, AF, and AT; in ECG he had low voltage P-waves. In Holter-monitoring, VES (600/24 h), SVES (1600/24 h), and NSVT were observed.

Another brother (II.9) was apparently healthy but died suddenly at the age of 24. He had no history of cardiac disease, syncope, or arrhythmias. An autopsy was performed, and histology showed significant fibrotic changes in epicardium and myocardium; there was no sign of inflammation. Another brother (II.10) died at the age of 34. He had congestive cardiomyopathy and suffered from AF, AFL, and NSVT. He had multiple SVES and VES in stress-test. Autopsy was performed and on histology, fibrofatty replacement of the myocardium was seen mainly in the septum and posterior wall, but some changes were seen also in both atria. No changes were observed in the right ventricle. Histological samples of brothers II.9 and II.10 were compared, and the findings were similar.

The proband’s father (I.1) died at the age of 88. On echocardiography, his LVEDD was 60 mm and LVEF was 64%. He suffered from AF and had right bundle branch block (RBBB) and left anterior hemiblock (LAHB) in ECG. The mother (I.2) died at the age of 80. On echocardiography, her LVEDD was 52 mm and LVEF was 52%. She had moderate mitral regurgitation and mild to moderate tricuspid-valve insufficiency. She suffered from AF and SVT, and, in Holter-monitoring, had findings suggestive of sick sinus syndrome.

Other relatives were also evaluated as a part of this study. One relative (II.1) had surgically repaired tetralogy of Fallot; cardiac findings were deemed to be associated with the postoperative state and congenital defect. All other relatives had normal findings in cardiac evaluations and were deemed unaffected.

## Discussion

In this study, we describe the cardiac phenotypes of six cardiomyopathy patients with homozygous truncating *GCOM1* variants from two families. All nine heterozygous individuals in the families were unaffected. The inheritance pattern is autosomal recessive. To our knowledge, neither of these *GCOM1* variants have been previously reported in the literature.

### GCOM1 and Human Cardiomyopathy

ClinVar does not contain any homozygous truncating variants in *GCOM1*; however, one truncating variant in one of the read through transcripts (*MYZAP*) is reported in a patient with DCM (ID: 523392) (March 2021).

All patients homozygous for either of the two *GCOM1* variants fulfilled the diagnostic criteria of DCM or ARVC. Some of these patients remained fairly asymptomatic even though the disease had advanced; for example, in Family one patient II.2 reported no symptoms but had suboptimal left ventricular function when primary cardiac evaluation was conducted. The phenotype became evident in both families by the age of 41; age of diagnosis did not vary greatly: the youngest was 24 years old and oldest was 41 at the time of diagnosis.

One deceased patient (Family 1, II.4) with a negative genotype fulfilled DCM imaging criteria but had histological findings compatible with lymphocytic myocarditis. Use of antipsychotic medication could have contributed to the phenotype. Although rare, quetiapine and risperidone have some level of associated risk with myocarditis and cardiomyopathy. (Coulter et al., 2001; Stoner, 2017). The EMB available was of poor quality, and we were not able to do further testing to confirm any other possible variants contributing to the patient’s cardiomyopathy. NGS panel testing was conducted on the parents, but no pathogenic/likely pathogenic or other promising variants in known cardiomyopathy genes were found.

Fluctuating TnI levels were seen in some patients over the years, troponin levels were quite high in some cases but normalized in follow-up. The relative lack of heart failure symptoms, young age, and high troponin levels could mislead clinicians in their primary diagnosis, especially if no family history is available. Myocarditis was sometimes suspected initially, but no sign of inflammation was observed in further examination. Inflammation could be related to the pathophysiology of the cardiomyopathy, but further studies are still needed to elucidate the background of these observations. In a small case report, reported that over half of their patients with clinically suspected myocarditis had a pathogenic or likely pathogenic variant in ARVC-associated genes. (Ader et al., 2020). They speculated that acute myocarditis could be the first clinical manifestation of inherited cardiomyopathy, especially arrhythmogenic cardiomyopathy. (Ader et al., 2020).

*GCOM1*/*MYZAP* variants have not been published as a cause of human cardiomyopathy, but at least one variant has been reported in association with atrial arrhythmias. In a meta-analysis of genome-wide association study, a *MYZAP* p. Gln254Pro missense variant was associated with increased risk of SSS and AF. (Thorolfsdottir et al., 2018). In our study, homozygotes seem to have a tendency for atrial arrhythmias. In 12-lead ECG, we noticed low amplitude P-waves in most of the precordial and extremity leads in homozygotes. The same was not observed in heterozygotes or family members not carrying the variant.

Five of the six homozygotes meet some cardiomyopathy criteria. Heart failure is the leading clinical feature, even though the patients may remain fairly asymptomatic. SCD was observed in one *GCOM1* homozygote, and post-mortem examination revealed fibrosis in the heart. The importance of the arrhythmogenic side of the phenotype and the risk of fatal arrhythmias remains unclear. Our findings confirm the *GCOM1* variants as a cause of human cardiomyopathy.

### Animal Models

*MYZAP,* one of the components in GRINL1A CTU, has been linked to cardiomyopathy in zebrafish and mice. (Seeger et al., 2010; Frank et al., 2014). Seeger *et al.* showed that knock-down of *MYZAP* in zebrafish results in cardiomyopathy with severe systolic dysfunction without significant cardiac enlargement or disturbing the cardiac development. (Seeger et al., 2010). Overexpression of Myozap, which is a protein encoded by *MYZAP*, caused cardiomyopathy with hypertrophy and LV enlargement in mice, and exercise accelerated these changes. (Frank et al., 2014). The cardiac phenotype in these mice mimicked desmin-related cardiomyopathies; even though desmin was not directly involved, protein aggregates containing Myozap and other intercalated disc (ID) proteins, such as desmoplakin, were present. (Frank et al., 2014).

### Genetic Background of DCM and ARVC

Classification of cardiomyopathies are mainly based on phenotypic and cardiac imaging criteria; for example in ARVC, the diagnosis is based on a set of complex criteria. (Elliott et al., 2008; Marcus et al., 2010; Corrado et al., 2020). Some overlap in phenotypic expression of cardiomyopathies can be seen, and overlap in genetic background has become evident as well. (Hershberger et al., 2013). The increasing amount of reported ARVC patients with greater LV involvement or biventricular disease has led to the proposition of arrhythmogenic cardiomyopathy. (Basso et al., 2012; Towbin et al., 2019). AC is a heterogeneous disorder associated with ventricular arrhythmias, systolic dysfunction, and increased risk of SCD. The estimated prevalence of AC ranges from 1:1,000 to 1:5,000. (Basso et al., 2012; Jacoby and McKenna, 2012). The lack of consensus criteria might lead to under-recognition of the disease.

Both ARVC and DCM are primarily inherited in an autosomal dominant pattern and there is some overlap in the genetic background. (Hershberger et al., 2010; Jacoby and McKenna, 2012; Campuzano et al., 2013; Hershberger et al., 2013; McNally et al., 2013). Autosomal recessive forms of both diseases are reported, although they are rarer. (Keller et al., 2012; Campuzano et al., 2013; McNally et al., 2013; McNally and Mestroni, 2017). The genetic etiology of DCM is broad and variants are identified in genes encoding sarcomeric proteins, cytoskeleton, ion channels, nuclear envelope, and intercellular junction proteins such as *TTN*, *LMNA, DSP, MYH7, FLNC, TNNT2, RBM20, DES, TPM1, FLNC,* and *DMD*. (Herman et al., 2012; McNally et al., 2013; Mestroni and Taylor, 2013; Akinrinade et al., 2015). Most ARVC/AC pathological variants are reported in genes encoding the proteins of the desmosome, including *PKP2, DSP, DSG2, JUP,* and *DSC2*. (Jacoby and McKenna, 2012; Campuzano et al., 2013). AC- and DCM-associated genes, such as *DSP*, *DES,* and *PKP2*, encode components of the ID. (Basso et al., 2012; Vermij et al., 2017). Remodeling of the ID has been shown in association with ARVC. (Basso et al., 2006).

The cardiac ID is a complex structure classically considered to consist of three main structures: desmosomes, adherens junctions, and gap junctions. Previously these three structures have been considered as individual units, but it has become evident that all components of the ID work as one functional unit. (Vermij et al., 2017). The main function of the ID is to attach adjacent cardiomyocytes and have the cardiac tissue work as a synchronized unit both mechanically and electrically. (Vermij et al., 2017). Because of its function, cardiac ID is considered to be in the center of pathophysiological mechanisms of cardiomyopathies. (Basso et al., 2006; Manring et al., 2018).

### Conjoined Genes

The GRINL1A CTU on chromosome 15q21.3-q22.1 is composed of two conjoined genes; the upstream centromeric group was originally referred to as Gup and later named *MYZAP,* and the downstream gene was referred to as Gdown, later named *POLR2M.* (Roginski et al., 2001; Roginski et al., 2004; Hu et al., 2006; Seeger et al., 2010). The CTU contains at least 28 exons that can be divided to three groups: upstream (exons 1–15), middle (exons 16–19), and downstream (exons 20–28). (Roginski et al., 2004). There are multiple start sites for transcription; upstream promoter (Pu) is located in exon one and downstream promoter (Pd) in exon 20. (Roginski et al., 2004). Pd transcription can initiate at two principal sites. (Roginski et al., 2004).

Transcription of the *POLR2M* initiates at the Pd and the transcripts contain only exons from the downstream group (Coulter et al., 2001; Seeger et al., 2010; Richards et al., 2015; Stoner, 2017; Thorolfsdottir et al., 2018; Ader et al., 2020; Decloaking Chamber, 2021; Peroxidase Blocking Solution, 2021; Rabbit IgG, 2021). *POLR2M* encodes the protein Gdown1, which is the 13^th^ subunit of a specific form of RNA polymerase II and acts as its suppressor. (Hu et al., 2006). *POLR2M* has not been shown to cause monogenic disease in humans.

*MYZAP* transcription initiates at the Pu, and the transcripts contain only certain exons from the upstream group ((Codd et al., 1989; Mestroni et al., 1999; Elliott et al., 2008; Hershberger et al., 2009; Hershberger et al., 2010; Marcus et al., 2010; Basso et al., 2012; Campuzano et al., 2013; Hershberger et al., 2013; McNally et al., 2013), (Towbin et al., 2019), (Corrado et al., 2020), and (Hershberger et al., 2018)). *MYZAP* encodes the Gup1 protein, also called Myozap (myocardium-enriched zona occludens-1-associated protein), which is highly conserved and strongly expressed in the myocardial tissue. (Seeger et al., 2010). In an immunohistochemical analysis of mouse cardiomyocytes, Myozap binds directly to desmoplakin and ZO-1, which are components of the ID. (Seeger et al., 2010). Myozap also localized in ID in immunoelectron microscopy of bovine heart. (Seeger et al., 2010). Yeast 2-hybrid analysis demonstrated that amino acids 91–250 of Myozap are required for binding to ZO-1. (Seeger et al., 2010).

Transcription of the *GCOM1* combined gene initiates at the Pu and then splices to middle or downstream exons, transcription is terminated at the downstream exons (23, 24, or 28). (Roginski et al., 2004). The combined transcripts include exons from all three groups; upstream exons from the *MYZAP* gene, downstream exons from the *POLR2M* gene as well as exons from the middle group (Roginski et al., 2001; Roginski et al., 2004; Hu et al., 2006; Roginski et al., 2018). (Roginski et al., 2004) Due to the inclusion of exons 16 and 19a and the use of different frames in downstream exons, *GCOM1* amino acid translations contain residues that are not present in neither *MYZAP* nor *POLR2M.* (Roginski et al., 2004).

Although the genetic background of cardiomyopathies has been under investigation for decades and much progress has been made, many patients undergoing genetic testing are still left without a genetic diagnosis. It is important to seek new candidate genes in addition to novel variants in known disease-causing genes when targeted gene panels do not yield a positive outcome, especially if familial cardiomyopathy is suspected. Since genetic testing uncovers a large number of variants at genome level, e.g., >30,000 variants per average exome, the analysis is typically limited to rare variants or less rare variant pairs when assuming dominant or recessive inheritance. In case of these CTU genes (*GCOM1*, *MYZAP*, *POLR2M*)*,* variants can be randomly called into different genes within the CTU, leaving the variant scientist or geneticist in a situation where they do not discover a variant pair due to an annotation issue. Thus, cardiomyopathy related to this CTU may be more common than discovered so far.

## Data Availability

The datasets presented in this article are not readily available because of the privacy of individuals that participated in the study and GDPR legislation. Requests to access the datasets should be directed to KH, krista.helio@helsinki.fi.
